# Efficacy of Liposomal Bupivacaine in Third Molar Extraction: A Systematic Review and Meta-Analysis

**DOI:** 10.7759/cureus.105390

**Published:** 2026-03-17

**Authors:** Mohammed Aljumaiaan, Abdulaziz Owayed, Ahmad Altourah, Yousef M Alawadhi, Abdulwahab Alkhamees, Mohamed Farid, Fadhel F. Alshammari, Abdullah Aldulaimi, Bedour Alhusainan, Layan Alameeri

**Affiliations:** 1 Department of Dentistry, Salwa Specialized Center Polyclinic, Ministry of Health, Kuwait City, KWT; 2 Department of Dentistry, Al-Amiri Hospital Dental Center, Ministry of Health, Kuwait City, KWT; 3 Department of Dentistry, Jaber Al-Ahmad 1 Polyclinic, Ministry of Health, Kuwait City, KWT; 4 Department of Dentistry, University of Sharjah, Sharjah, ARE; 5 Department of Dentistry, Al-Rawda Healthcare Center, Ministry of Health, Kuwait City, KWT; 6 Faculty of Dentistry, Modern Science and Arts University, Giza, EGY; 7 Department of Dentistry, Farwaniyah Polyclinic, Ministry of Health, Kuwait City, KWT; 8 Department of Dentistry, Ministry of Health, Kuwait City, KWT; 9 Department of Dentistry, Hiteen Dental Center, Mubarak Al-Hassawi Hospital, Ministry of Health, Kuwait City, KWT

**Keywords:** liposomal bupivacaine, meta-analysis, opioid-sparing, postoperative pain, third molar, wisdom tooth

## Abstract

Third molar extraction is a common surgical procedure, and it is associated with significant postoperative pain. Liposomal bupivacaine (LB) is an extended-release local anesthetic. This intervention was developed in its current form to provide prolonged analgesia and reduce opioid use. However, its efficacy in third molar surgery remains inconclusive and inconsistent. In this systematic review and meta-analysis, we aim to evaluate the efficacy of LB in third molar extraction. We systematically searched PubMed, Scopus, Web of Science, and the Cochrane Library up to January 2026 for randomized controlled trials (RCTs) and observational studies comparing LB to standard bupivacaine or placebo. We performed the meta-analysis using R software with a random-effects model. Continuous outcomes were assessed using mean differences (MDs) or standardized mean differences (SMDs) with 95% confidence intervals (CIs). Four studies (three RCTs, one observational) with 858 participants were included. LB showed a statistically significant but clinically negligible reduction in opioid consumption at 48 hours (MD −1.20 morphine milligram equivalents (MME); 95% CI −1.79 to −0.61). One study reported a substantial reduction in opioid prescriptions (MD −66.70 MME). For analgesia, there was no statistically significant difference between LB and control in postoperative pain intensity (SMD −0.21; 95% CI −0.51 to 0.10) or cumulative pain scores (SMD −1.06; 95% CI −2.52 to 0.39) over 0-48 hours, with high heterogeneity in the latter (*I*² = 96.9%). Our findings indicate that while LB was associated with a significant reduction in opioid consumption, the effect size is not clinically meaningful, with a limited difference in postoperative pain. However, the limited number of included studies highlights the need for cautious interpretation, with further large-scale RCTs needed to validate these findings.

## Introduction and background

Third-molar surgical extraction is a common dental procedure, with up to 5 million patients undergoing extraction of nearly 10 million teeth in the United States per year [1,2]. This procedure is associated with a significant inflammatory response and is characterized by acute postoperative pain that typically peaks at 6-12 hours and may persist for three to seven days, impairing oral function and delaying daily activities [3,4]. The management of this pain has heavily relied on opioids. Data indicate that dental-related pain remains the second most frequent cause of immediate-release opioid prescriptions, accounting for 15.8% of all prescriptions [5,6]. However, this presents a public health concern, as this initial opioid exposure for adolescents and young adults may increase the risk of persistent use and misuse [7]. 

To manage these concerns, clinical practice has increasingly been interested in non-opioid analgesic strategies, with the current protocols emphasizing nonsteroidal anti-inflammatory drugs (NSAIDs)/acetaminophen combination as the primary pharmacologic choices [8]. However, breakthrough pain remains prevalent, and it may require opioids [9]. The use of long-acting local anesthetics (e.g., 0.5% bupivacaine hydrochloride with epinephrine (standard bupivacaine) was also presented as a component of a multimodal framework to reduce postoperative discomfort [10,11]. However, standard bupivacaine’s efficacy is limited; its duration of action typically does not exceed eight hours [10,11]. This short window is often insufficient and does not cover the gap between surgical anesthesia and the peak of inflammation.

For these temporal limitations, liposomal bupivacaine (LB) was developed as a prolonged-release multivesicular liposome-encapsulated bupivacaine formulation, which received U.S. Food and Drug Administration (FDA) approval in 2011 [12]. LB uses a proprietary DepoFoam technology, which encapsulates the drug within a honeycomb-like structure of multivesicular liposomes [13]. This delivery system facilitates a bimodal release profile: an initial peak of free bupivacaine, then a sustained release as the lipid matrix degrades [13]. This mechanism could extend the analgesic duration to 72-96 hours through a single administration, covering the window of peak postoperative pain, and it may potentially reduce the use of systemic analgesics [14].

However, the clinical evidence regarding LB in third molar extraction remains inconsistent, with different outcomes across studies. The INNOVATE trial compared LB to placebo; although it did not meet the primary efficacy endpoint in the intent-to-treat population, a per-protocol analysis suggested a reduction in pain [15]. Lieblich et al. provided strong real-world evidence, reporting a 59% reduction in prescribed morphine milligram equivalents and significantly lower refill rates in the LB cohort [16]. Newer randomized controlled trials (RCTs) encompassing active comparators have challenged these findings. James et al.’s RCT found no statistically significant difference in postoperative pain or narcotic consumption at 48 hours when comparing LB to standard bupivacaine [17]. More recently, Young et al. observed only a modest reduction in pain with LB [18]. Therefore, it remains unclear whether LB can achieve the above-mentioned theoretical benefits in third molar extraction. To date, a comprehensive quantitative synthesis addressing this question is lacking. Therefore, we conducted this systematic review and meta-analysis to evaluate the efficacy of LB infiltration use in third molar extraction. 

## Review

Methodology

Study Registration

The study protocol was registered on PROSPERO (CRD420261297642). This systematic review adhered to the Preferred Reporting Items for Systematic Reviews and Meta-Analyses (PRISMA) statement [19] and was conducted in accordance with the guidelines of the Cochrane Handbook for Systematic Reviews of Interventions [20]. 

Literature Search and Study Selection

We searched PubMed, Scopus, Web of Science, and Cochrane Library databases from inception up to January 2026 using search terms related to "third molar," "wisdom tooth," "tooth extraction," and "liposomal bupivacaine" (Table 1). 

**Table 1:** Search strategies.

Databases	Search strategies	Results	Limitations	Date of search
PubMed	("molar surgery" OR "third molar" OR "wisdom tooth" OR "wisdom teeth" OR "tooth extraction" OR "dental extraction" OR dentoalveolar OR "odontectomy" OR "mandibular third molar") AND ("liposomal bupivacaine" OR "Exparel" OR "bupivacaine liposome" OR "LB" OR "extended-release bupivacaine" ) (All fields)	48	No limitations applied	January 10, 2026
Web of Science	("molar surgery" OR "third molar" OR "wisdom tooth" OR "wisdom teeth" OR "tooth extraction" OR "dental extraction" OR dentoalveolar OR "odontectomy" OR "mandibular third molar") AND ("liposomal bupivacaine" OR "Exparel" OR "bupivacaine liposome" OR "LB" OR "extended-release bupivacaine" ) (All fields)	89	No limitations applied	January 10, 2026
Cochrane Library	("molar surgery" OR "third molar" OR "wisdom tooth" OR "wisdom teeth" OR "tooth extraction" OR "dental extraction" OR dentoalveolar OR "odontectomy" OR "mandibular third molar") AND ("liposomal bupivacaine" OR "Exparel" OR "bupivacaine liposome" OR "LB" OR "extended-release bupivacaine" ) (All text)	23	No limitations applied	January 10, 2026
Scopus	TITLE-ABS-KEY("molar surgery" OR "third molar" OR "wisdom tooth" OR "wisdom teeth" OR "tooth extraction" OR "dental extraction" OR dentoalveolar OR "odontectomy" OR "mandibular third molar" ) AND ("liposomal bupivacaine" OR "Exparel" OR "bupivacaine liposome" OR "LB" OR "extended-release bupivacaine")	34	No limitations applied	January 10, 2026

We used EndNote software (Clarivate Analytics, Philadelphia, PA) to remove duplicates [21]. We uploaded the retrieved references to the Rayyan website [22], and two independent authors conducted a two-step screening process: the first step involved screening titles and abstracts, and the second step involved screening the full-text articles of the identified abstracts for final eligibility. Any conflicts were resolved by a third author.

Eligibility Criteria

We included studies meeting the following PICO criteria: Population: Adult patients (≥18 years) undergoing third molar extraction; Intervention: LB; Control: Standard bupivacaine or placebo; Outcomes: Postoperative pain, opioid use or prescription; Study design: RCTs and observational studies.

We excluded reviews, in vitro studies, animal studies, non-English studies, and non-peer-reviewed articles.

Data Extraction

Two independent authors extracted data using a standardized Excel sheet, including study characteristics (design, sample size, intervention, control, follow-up, outcomes), baseline characteristics (age, gender, ethnicity), and outcome measurements.

Quality Assessment

Two independent authors evaluated the risk of bias in included RCTs using the Cochrane Risk of Bias (ROB)-2 assessment tools [23]. This evaluation encompassed an assessment of the randomization process, concealment of the allocation sequence, deviations from the intended interventions, utilization of appropriate analysis to estimate the effect of assignment to intervention, measurement of the outcome, selection of the reported results, and overall risk of bias. However, regarding the cohort study, we used the Newcastle-Ottawa Quality assessment form for cohort studies, which encompassed an assessment of three domains: selection, comparability, and outcome [24]. Any conflict was resolved by a third author. The Robvis web tool was used to create quality assessment figures [25]. 

Statistical Analysis

We used R 4.5.0 [26] with R Studio 2024.12.1+563 [27]. Continuous data were analyzed using mean difference (MD), standardized mean difference (SMD), and 95% confidence intervals (CIs). Visual inspection of the forest plot was used to measure statistical heterogeneity between trials, in addition to I-squared (I^2^) and chi-squared (Chi^2^) statistics. *I*^2^ values of 50% indicated significant heterogeneity. Publication bias and small-study effects assessment were not feasible due to the limited number of included studies [28]. All results were analyzed using a random-effects model. 

Results

Literature Search

**Figure 1:**
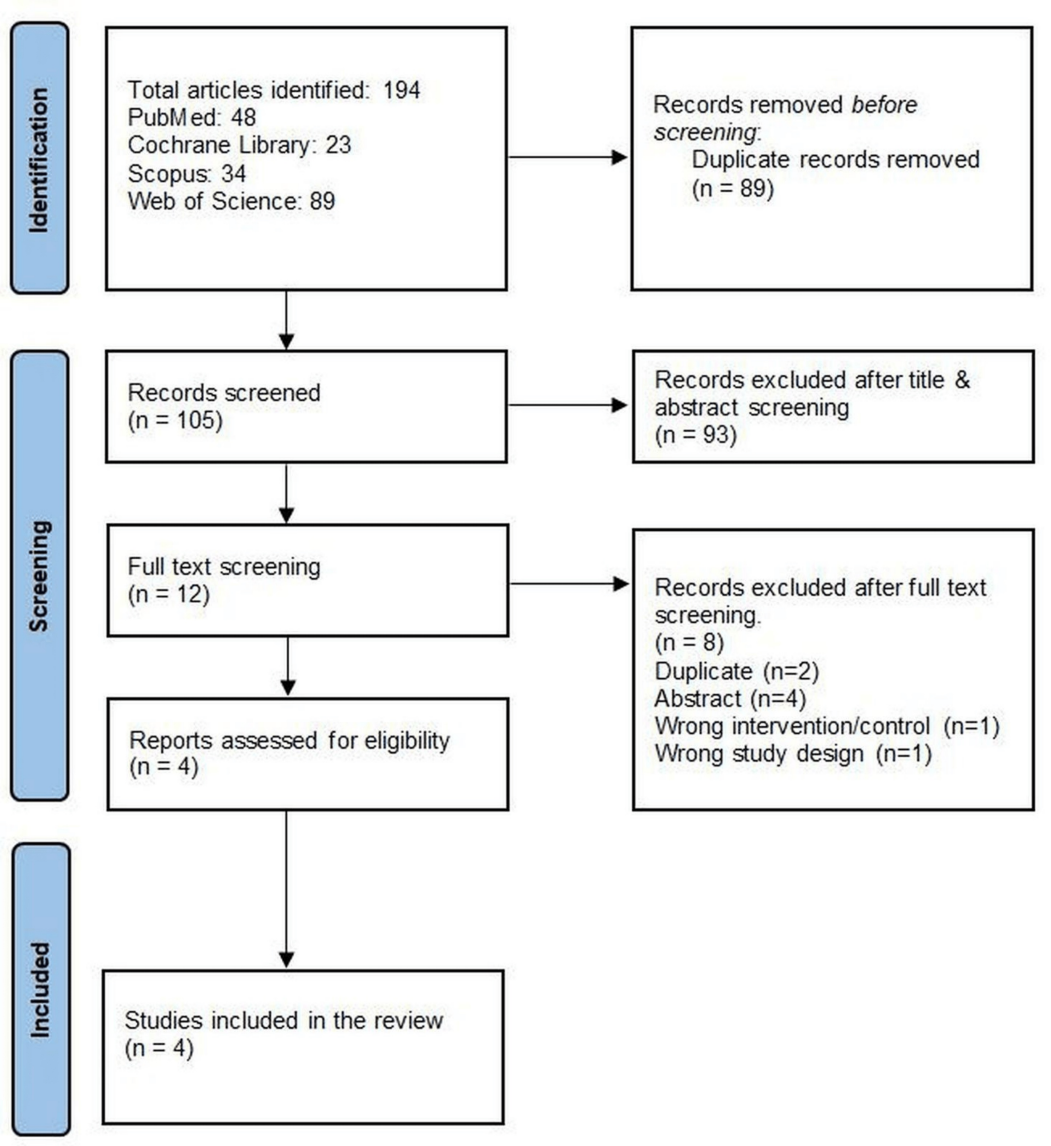
PRISMA flowchart. [15-18]. PRISMA, Preferred Reporting Items for Systematic Reviews and Meta-Analyses

Our search identified 194 records. After removing 89 duplicates, 105 records were screened by title and abstract. Of these, 12 full-text articles were assessed. Finally, three RCTs and one observational study were included in this review and analysis [15-18]. The PRISMA flow diagram is shown in Figure 1.

Study Characteristics

Four studies with 858 participants were included [15-18]. The pooled sample consisted of 417 patients in the LB group and 369 in the control group, with 72 additional patients contributing to both arms in a split-mouth RCT. Mean age ranged from 20.9 to 25.8 years, and male participants comprised 16% to 50% of the study populations. Preoperative pain levels varied across studies. Study details are provided in Tables 2-3.

**Table 2:** Summary of included studies. RCT, randomized controlled trial; ASA, American Society of Anesthesiologists; VAS, visual analog scale; NRS, numeric rating scale; AUC, area under the curve; LA, local anesthesia; ITT, intention-to-treat

Study ID	Study design	Country	Total sample size, no.	Population	Inclusion criteria	Intervention	Control	Follow-up period, weeks	Primary outcomes
James et al., 2022 [17]	Parallel-arm RCT	United States	24 (Analyzed)	Patients (18-45 years) undergoing extraction of bilateral impacted mandibular third molars, ASA I-II.	Age 18-45 years, bilateral impacted mandibular 3rd molars, specific impaction types (uncomplicated mesioangular or vertical), ASA I-II.	Liposomal bupivacaine infiltration (LBI): 1 mL (0.5 mL x 2 points) per extraction site.	Standard bupivacaine: 0.25% bupivacaine with 1:200k epinephrine, same infiltration method (1 mL per site).	48 hours-1 week	Postoperative pain levels at 48 hours and 1 week, measured via VAS (0-10).
Lieblich and Danesi, 2017 (INNOVATE) [15]	Phase 3, Double-blind, Placebo-controlled RCT	United States	150 (Primary Efficacy/ITT)	Subjects ≥18 years undergoing bilateral 3rd molar extraction (all 4 molars), with ≥1 mandibular molar partial/full bony impaction, ASA I-III.	≥18 years, bilateral 3rd molar extraction under LA, ≥1 mandibular bony impaction.	Liposomal bupivacaine (LB): 133 mg/10 mL infiltrated (4 mL maxilla, 6 mL mandible).	Placebo: Sterile normal saline (10 mL), infiltrated identically.	96 hours (4 days) + follow-up calls to day 30	Cumulative pain intensity through 48 hours (AUC of NRS 0-48).
Lieblich et al., 2021 [16]	Retrospective study	United States	600	Patients ≥18 years undergoing extraction of ≥1 partial or full bony impacted mandibular 3rd molar at two outpatient centers.	Extraction of ≥1 partial/full bony impacted mandibular 3rd molar.	Liposomal bupivacaine (LB): 133 mg/10 mL infiltration.	Non-LB group: Standard care without liposomal bupivacaine.	48 hours	Total prescribed opioids in Morphine Milligram Equivalents (MMEs).
Youn et al., 2025 [18]	Double-blind, Split-mouth RCT	United States	72	Subjects undergoing bilateral mandibular 3rd molar extractions.	Bilateral mandibular 3rd molar extractions. Excluded: additional mandibular extractions, inability to complete questionnaire, specific medical contraindications.	Liposomal bupivacaine (LB): 3 mL (39.9 mg) infiltrated per side.	Standard bupivacaine (SB): 0.5% bupivacaine diluted (3 mL, 10 mg) infiltrated per side.	96 hours (4 days)	Postoperative pain was measured via NRS (0-10) and cumulative pain (AUC NRS 0-96).

**Table 3:** Baseline characteristics of study participants. SD, standard deviation; LB, liposomal bupivacaine; SB, standard bupivacaine

Study ID	Study group	Sample size, *n*	Age, mean (SD)	Male, *n* (%)	Caucasian/White	African American	Asian	Other races
James et al., 2022 [17]	Liposomal bupivacaine (LB)	12	22.9 (±5.4)	2 (16.7%)	8 (66.7%)	4 (33.3%)	0 (0%)	0 (0%)
	Control (SB)	12	25.4 (±6.2)	6 (50.0%)	10 (83.3%)	1 (8.3%)	1 (8.3%)	0 (0%)
Lieblich and Danesi, 2017 (INNOVATE) [15]	LB	105 (safety population)	20.9 (±3.8)	52 (49.5%)	94 (89.5%)	9 (8.6%)	Not reported	2 (1.9%)
	Control (Placebo)	57 (safety population)	20.9 (±4.8)	24 (43.6%)	53 (93.0%)	1 (1.8%)		3 (5.3%)
Lieblich et al., 2021 [16]	LB	300	22.4	125 (41.7%)	99 (33.0%)	36 (12.0%)	1 (0.3%)	14 (4.7)
	Control (Non-LB)	300	23.5	134 (44.7%)	71 (23.7%)	53 (17.7%)	4 (1.3%)	22 (7.3)
Youn et al., 2025 [18]	LB	72	25.8 (±9.0)	35 (48.6%)	Not reported	Not reported	Not reported	Not reported
	Control (SB)							

Quality Assessment

**Figure 2:**
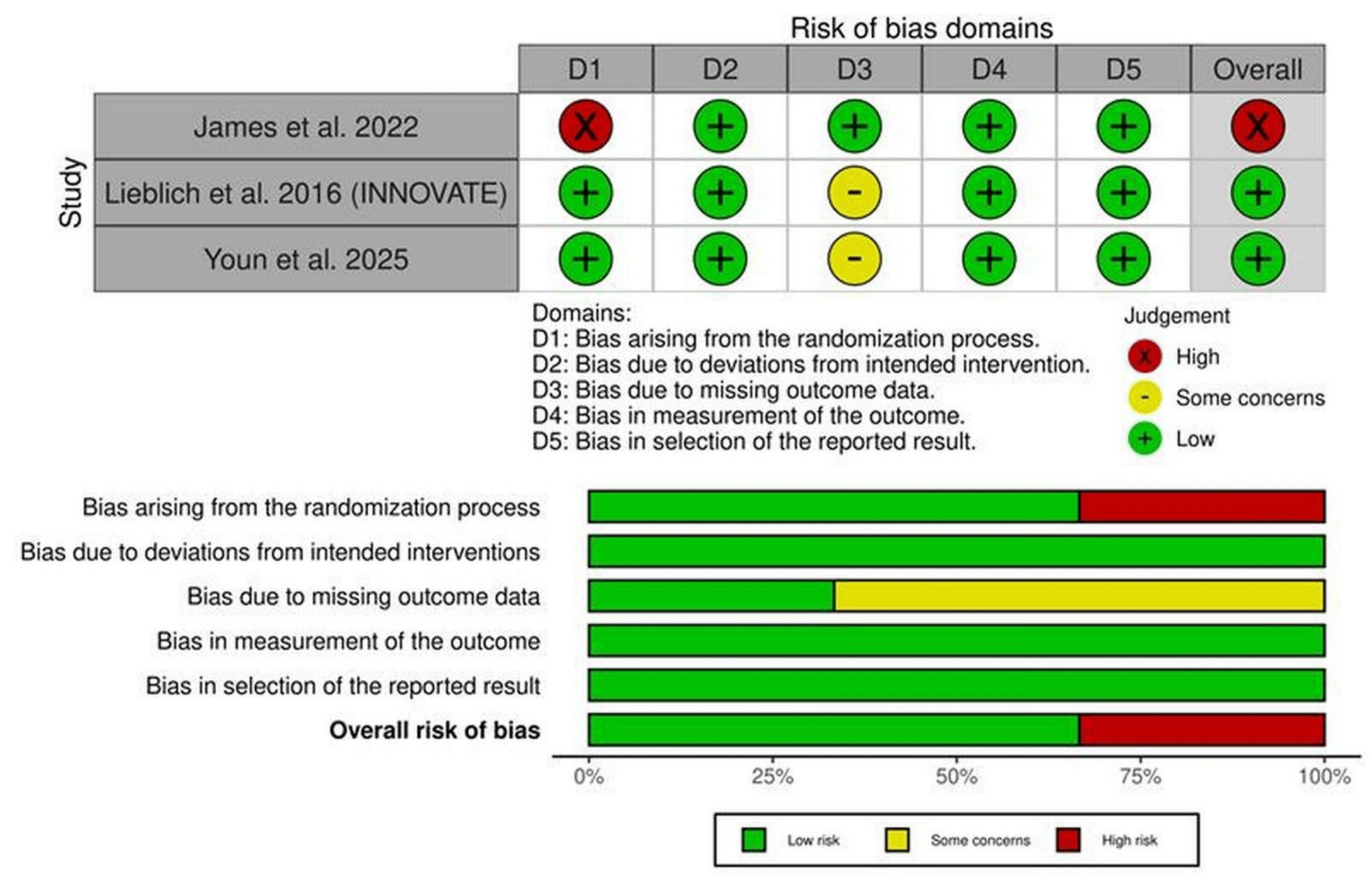
Summary and traffic-light plot of risk of bias in randomized controlled trials (RCTs). [15,17,18]

Using the ROB2 tool, two studies were rated as having an overall low risk of bias [15,18], while one study had a high risk of bias [17]. The risk of bias assessment is summarized in Figure 2. For the retrospective study [16], it had a total score of 8, indicating good quality across the assessed domains (Table 4).

**Table 4:** Newcastle-Ottawa Quality Assessment for retrospective studies. [16]

Domain	Criteria	Lieblich et al. (2021)
Selection	(1) Representativeness of the exposed cohort	★
	(2) Selection of the non-exposed cohort	★
	(3) Ascertainment of exposure	★
	(4) Outcome not present at start of study	★
Comparability	(5) Comparability of cohorts (design/analysis)	★
Outcome	(6) Assessment of outcome	★
	(7) Follow-up long enough for outcomes	★
	(8) Adequacy of follow-up of cohorts	★
Total Score		8 (Good quality)

Efficacy Outcomes

Primary efficacy outcomes: Opioid outcomes at 48 hours

**Figure 3:**
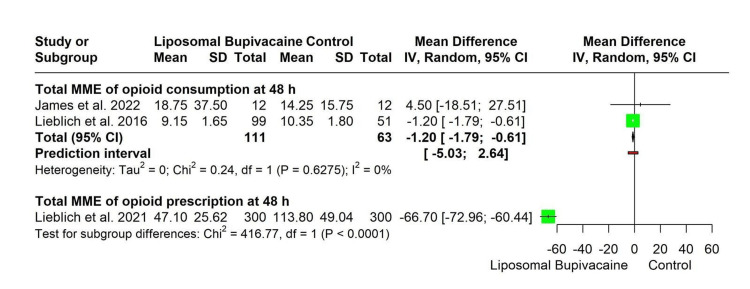
Forest plot for subgroup analysis of Opioid Outcomes at 48 Hours [15-17]

Total morphine milligram equivalent (MME) of opioid consumption at 48 hours: Two studies involving a total of 174 participants (LB: *n* = 111; control: *n* = 63) were included in the meta-analysis of opioid consumption at 48 hours. Using a random-effects model, LB was associated with a statistically significant reduction in opioid consumption compared with control (MD −1.20 MME; 95% CI −1.79 to −0.61). There was no evidence of statistical heterogeneity among studies (τ² = 0; χ² = 0.24, df = 1, *P* = 0.63; *I*² = 0%). The prediction interval ranged from −5.03 to 2.64 MME, indicating potential variability in the magnitude of effect across future studies (Figure 3).

Total MME of opioid prescription at 48 hours: One study, including 600 participants (LB: *n* = 300; control: *n* = 300), evaluated total opioid prescription at 48 hours. LB was associated with a substantial reduction in prescribed opioids compared with control (MD −66.70 MME; 95% CI −72.96 to −60.44) (Figure 3).

Subgroup differences: A prespecified subgroup analysis comparing opioid consumption and opioid prescription outcomes demonstrated a statistically significant difference between subgroups (χ² = 416.77, df = 1, *P* < 0.0001), indicating that the magnitude of the effect of LB differed significantly depending on the opioid outcome assessed (Figure 3). 

Postoperative Pain Intensity at 0-48 Hours

**Figure 4:**
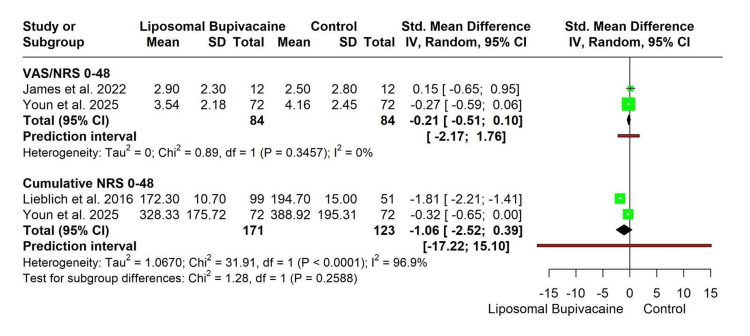
Forest plot for subgroup analysis of postoperative pain intensity at 0-48 hours. [15,17,18]

VAS/NRS scores at 0-48 hours: Two studies, including a total of 168 participants (LB: *n* = 84; control: *n* = 84), reported postoperative pain intensity using VAS or NRS scales during the first 48 hours. Using a random-effects model, there was no statistically significant difference between LB and control (SMD −0.21; 95% CI −0.51 to 0.10). There was no evidence of heterogeneity between studies (τ² = 0; χ² = 0.89, df = 1, *P* = 0.35; *I*² = 0%). The prediction interval ranged from −2.17 to 1.76, indicating substantial uncertainty in the expected effect size in future studies (Figure 4).

Cumulative NRS scores at 0-48 hours: Two studies involving 294 participants (LB: *n* = 171; control: *n* = 123) evaluated cumulative pain scores over the first 48 hours. The pooled analysis demonstrated no statistically significant reduction in cumulative pain with LB compared with control (SMD −1.06; 95% CI −2.52 to 0.39). This analysis showed considerable heterogeneity (τ² = 1.07; χ² = 31.91, df = 1, *P* < 0.0001; *I*² = 96.9%). The prediction interval was wide (−17.22 to 15.10), reflecting substantial variability in effect estimates across studies (Figure 4).

Subgroup differences: There was no statistically significant difference between the VAS/NRS 0-48 hours and cumulative NRS 0-48 hours subgroups (χ² = 1.28, df = 1, *P* = 0.26), suggesting that the effect of LB on postoperative pain did not differ significantly according to pain measurement approach (Figure 4). 

Discussion

Summary of Findings

The analysis findings regarding opioid outcomes at 48 hours; LB demonstrated a statistically significant reduction in opioid consumption compared to controls (MD -1.20 MME; 95% CI -1.79 to -0.61). However, the magnitude of this reduction is clinically negligible. Our analysis of opioid prescription volumes revealed a statistical reduction (MD -66.70 MME). For analgesia, our pooled analysis found no statistically significant difference in postoperative pain intensity between LB and controls in the first 48 hours (VAS/NRS SMD -0.21) or the cumulative NRS (SMD -1.06). However, the cumulative pain analysis exhibited substantial heterogeneity (*I*^2^ = 96.9%). 

Interpretation of Findings

The postsurgical pain related to third molar extraction presents a challenge in balancing patient comfort against opioid stewardship. LB has been introduced to reduce the need for systemic narcotics, and this meta-analysis synthesized available data regarding its efficacy. These findings challenge the narrative regarding the role of LB in oral and maxillofacial surgery (OMS), particularly third molar extraction. Our results did not reveal convergence between the assumed pharmacokinetic benefits (DepoFoam multivesicular liposome technology) [29] and the actual clinical performance, though cautious interpretation is needed given the limited number of included studies.

For the analgesic efficacy, the primary analysis demonstrated that LB provided no statistically significant advantage in postoperative pain intensity over controls during the acute (0-48 hours) window (SMD -0.21). This aligns with and confirms the results of the active-comparator RCT conducted by James et al. [17], who reported that LB, when compared directly to 0.5% standard bupivacaine, was not characterized by a statistically significant benefit regarding pain levels at any collected time point [17]. However, James et al. did note a significant difference in adjusted pain levels at the one-week follow-up, although the authors themselves questioned the clinical relevance of such a delayed finding [17]. Our meta-analysis aggregate confirms this; the prolonged-release formulation did not translate to superior analgesia in the critical acute phase. 

On the other hand, the Phase 3 INNOVATE trial by Lieblich and Danesi [15] reported significantly lower pain intensity in patients receiving LB; however, this trend was noted only in the per-protocol population [30]. As highlighted by Kramer, the INNOVATE study failed to meet its primary endpoint in the intent-to-treat (ITT) population due to many protocol violations, necessitating the per-protocol differentiation [30]. By pooling ITT data, our meta-analysis is conservative regarding the potential confounders, reflecting a more realistic estimation of the drug's performance. For the most recent study (the split-mouth RCT by Youn et al.), which reported a statistically significant reduction in pain (particularly in bilateral extractions) favoring LB, the magnitude of this reduction was characterized as modest, with differences ranging only from 0.08 to 0.98 on a 10-point scale [18]. The high heterogeneity observed in our cumulative pain analysis (*I*^2^ > 96%) suggests that this benefit may be dependent on surgical variables; LB may offer marginal superiority only in cases of extreme surgical trauma where standard local anesthetics reach a ceiling effect. For routine impactions, however, the data showed that the addition of the liposomal matrix did not provide meaningful analgesic benefit over standard regimens.

In the broader landscape of OMS literature [29], the use of LB in different procedures yielded a transient nature of benefits. For instance, in full-arch implant surgery, patients treated with LB showed significantly greater satisfaction with pain control, but only for the first 24 hours postoperatively [31]. Similarly, in pediatric palatoplasty and pharyngoplasty, significantly lower pain scores were observed only in the immediate postoperative period [32]. Investigations into post-tonsillectomy analgesia and buccal mucosal graft harvests yielded similar results: pain reduction and decreased opioid use were statistically significant only on the first postoperative day, with no difference thereafter [33,34]. These comparative studies and our findings point to the fact that the 72- to 96-hour duration of action claimed by the manufacturer [14,30] is rarely realized in the highly vascular, anatomically constrained oral cavity. The alignment between our third molar data and these diverse surgical models reinforces the conclusion that standard multimodal analgesic protocols, likely combined with standard amide anesthetics, are sufficient (or at least non-inferior) to manage the bulk of acute postsurgical pain without the need for liposomal adjuncts.

We observed a statistically significant but clinically negligible reduction in opioid consumption (-1.20 MME). This absolute lack of a meaningful consumption reduction confirms James et al.’s findings, who found no significant difference in the number of narcotic pills taken between LB and standard bupivacaine groups [17]. Furthermore, we align with the comprehensive systematic review by Ji et al., which analyzed 77 RCTs and found that LB did not show a reduction in opioid consumption in 85.71% of RCTs, yielding no reduction in opioid use in 83.33% of studies when compared to standard bupivacaine [35].

However, we also observed a reduction in opioid prescriptions (-66.70 MME) with LB. This finding was driven by Lieblich et al.’s retrospective study, which showed a 59% reduction in prescribed MMEs in patients receiving LB [16]. This observation introduces a paradox, where prescriptions drop dramatically but actual consumption remains unchanged compared to controls. As Kramer noted, Lieblich et al.'s retrospective protocol included scheduled ibuprofen but a suboptimal, breakthrough-only acetaminophen regimen, leading to potential confounding [30]. More importantly, the reduction in prescriptions might be a behavioral phenomenon, not a pharmacological one. Dental surgeons who inject LB anticipate 96 hours of analgesia and therefore proactively reduce their initial narcotic prescriptions. This may demonstrate that the LB’s opioid-sparing effect may be related to a shift in prescriber confidence and the implementation of contemporary ERAS protocols, rather than a drug advantage [30].

We expand the results by Ji et al. regarding the OMS LB’s literature [35]. Ji et al. reported that clinical trials with a financial conflict of interest relating to the manufacturer of LB were significantly more likely to show pain relief (odds ratio (OR) = 14.31, *P* = 0.0001) and decreased opioid consumption (OR = 12.35, *P* = 0.0237) [35]. Additionally, the clinical benefit should be weighed against the socioeconomic cost. A 10-mL vial of Exparel costs roughly $225, an expense that most dental insurance and state Medicaid plans do not cover [30]. In addition, in their cross-sectional study, Ho et al. demonstrated that while 76% of patients were interested in LB, 88% of patients had a willingness-to-pay threshold below $200 [36]. While interest in LB was associated with patients concerned about the addictive potential of opioids (aOR = 4.04), the out-of-pocket cost remains a barrier to acceptability [36].

Strengths and Limitations

To our knowledge, this is the first review to assess the efficacy of LB in third molar extraction. Our study has some strengths, such as employing random-effects models with prediction intervals and subgroup testing. Additionally, the differentiation between opioid prescription and consumption, which unveils the behavioral versus pharmacological components of the opioid-sparing effect. However, several limitations must be acknowledged. The small study numbers and the wide prediction intervals reduce precision despite statistical significance. In our analysis, there was high statistical heterogeneity observed in cumulative pain scores. This observation can be attributed to variability in surgical difficulty and studies’ protocols. The outcome regarding opioid prescription relied on a single retrospective study, which carries inherent selection biases and confounding; however, this was the only available study that assessed this outcome.

Clinical Implications

The routine use of LB for uncomplicated third molar extractions should be carefully reconsidered, particularly given its high-cost relative to standard bupivacaine. Future research is needed and should prioritize large-scale, independent RCTs comparing LB versus optimized standard of care regimens.

## Conclusions

While LB provides a statistically significant reduction in opioid consumption, the effect size is clinically irrelevant. While LB showed limited clinically meaningful superiority for postoperative pain control or actual opioid consumption in third molar surgery, caution is needed given the limited number of included studies. Further, larger transparent RCTs are needed to support its role.
